# Towards a new tuberculosis drug: pyridomycin – nature's isoniazid

**DOI:** 10.1002/emmm.201201689

**Published:** 2012-09-17

**Authors:** Ruben C Hartkoorn, Claudia Sala, João Neres, Florence Pojer, Sophie Magnet, Raju Mukherjee, Swapna Uplekar, Stefanie Boy-Röttger, Karl-Heinz Altmann, Stewart T Cole

**Affiliations:** 1 Ecole Polytechnique Fédérale de Lausanne, Global Health InstituteLausanne, Switzerland; 2 Eidgenössische Technische Hochschule Zürich, Institut für Pharmazeutische WissenschaftenHCI H 405, Zürich, Switzerland

**Keywords:** drug discovery, InhA, isoniazid, pyridomycin, tuberculosis

## Abstract

Tuberculosis, a global threat to public health, is becoming untreatable due to widespread drug resistance to frontline drugs such as the InhA-inhibitor isoniazid. Historically, by inhibiting highly vulnerable targets, natural products have been an important source of antibiotics including potent anti-tuberculosis agents. Here, we describe pyridomycin, a compound produced by *Dactylosporangium fulvum* with specific cidal activity against mycobacteria. By selecting pyridomycin-resistant mutants of *Mycobacterium tuberculosis*, whole-genome sequencing and genetic validation, we identified the NADH-dependent enoyl- (Acyl-Carrier-Protein) reductase InhA as the principal target and demonstrate that pyridomycin inhibits mycolic acid synthesis in *M. tuberculosis*. Furthermore, biochemical and structural studies show that pyridomycin inhibits InhA directly as a competitive inhibitor of the NADH-binding site, thereby identifying a new, druggable pocket in InhA. Importantly, the most frequently encountered isoniazid-resistant clinical isolates remain fully susceptible to pyridomycin, thus opening new avenues for drug development.

→ See accompanying article http://dx.doi.org/10.1002/emmm.201201811

## INTRODUCTION

Today, infection with *Mycobacterium tuberculosis* accounts for up to two million deaths annually (Glaziou et al, 2009). Major confounding factors such as poverty, homelessness and the prevalence of HIV/AIDS (Harrington, 2010) mean that tuberculosis will indefinitely remain an important cause of morbidity and mortality throughout the world. Furthermore, despite the small, but growing number of drugs that are effective at killing *M. tuberculosis*, the current treatment is still burdened by its duration (typically 6 months for drug-sensitive strains) and the ever increasing number of multidrug (MDR) and extensively drug resistant (XDR) clinical isolates of *M. tuberculosis* (Cegielski, 2010). Together, this underlines the need for alternative therapeutic entities that can be used both to shorten the duration of therapy and to combat the growing problem of clinical drug resistance.

Natural products have long provided a rich source of effective anti-tuberculosis agents. The most active of these in current use, the rifamycins (rifampicin, rifabutin and rifapentine), inhibit RNA polymerase and are crucial for front-line treatment of the disease. Furthermore, several other natural products such as the aminoglycosides (streptomycin, amikacin and kanamycin) and the peptide antibiotic (capreomycin) are part of the current portfolio of anti-tuberculosis drugs. The rich diversity of natural products represents a powerful tool for drug discovery, firstly, in the form of leads for potential anti-microbial agents and secondly, as a means of identifying those targets that are most vulnerable in the bacterium.

**Figure 1:**
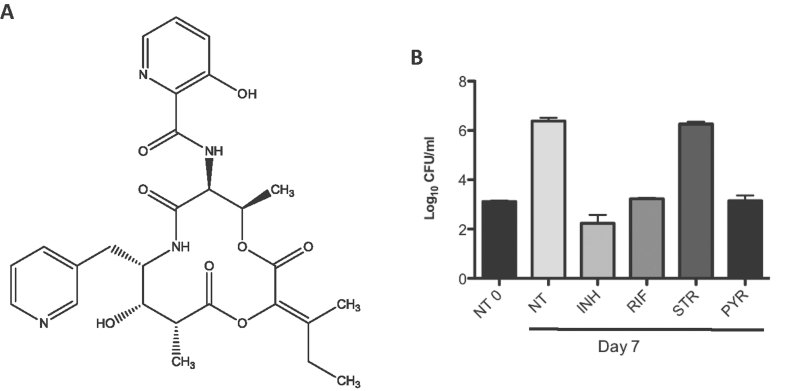
Chemical structure and intracellular activity of pyridomycin Chemical structure of pyridomycin.The activity of pyridomycin on intracellular *M. tuberculosis* was tested in activated THP-1-derived macrophages. Cells were infected at an MOI of 1:1 with *M. tuberculosis* Erdman and treated with isoniazid (INH) at 1 µg/ml, rifampicin (RIF) at 1 µg/ml, streptomycin (STR) at 10 µg/ml or pyridomycin (PYR) at 10 µg/ml. Colony forming units (CFU) were determined after 7 days exposure to drugs. NT refers to the untreated sample and NT0 to untreated sample at time 0. The experiment was performed in duplicate and results are shown as mean values and standard errors.

In 1953, pyridomycin was first described as an antibiotic that exhibited specific activity against different mycobacteria including *M. tuberculosis* and *M. smegmatis* (Maeda et al, 1953). Pyridomycin (Fig 1A) is produced by *Streptomyces pyridomyceticus* (Maeda et al, 1953; Yagishita, 1954, 1955, 1957a, b) or *Dactylosporangium fulvum* (Shomura et al, 1986). Its biosynthesis was first studied in 1968 (Ogawara et al, 1968) and more recently in 2011 (Huang et al, 2011) when the involvement of both non-ribosomal peptide synthetases (NRPS) and polyketide synthases (PKS) was proposed. Despite this body of work, the mechanism of action of pyridomycin against *M. tuberculosis* is unknown, and its potential as an anti-tuberculosis compound has not been assessed.

The aim of this study was to determine how pyridomycin kills *M. tuberculosis* and to identify its target. To achieve this, a combination of approaches involving resistance mapping, genetic validation, biochemistry, enzyme inhibition and X-ray crystallographic analysis of the target are described. The combined results unambiguously indicate that pyridomycin is a competitive inhibitor of the NADH-binding site of InhA, NADH-dependent enoyl-[Acyl-Carrier-Protein] reductase, the target of the two anti-tuberculosis pro-drugs isoniazid and ethionamide (Banerjee et al, 1994; Vilcheze et al, 2006).

## RESULTS

### Purification of pyridomycin

Several strains of *Streptomyces pyridomyceticus* (NRRL B-2517, ISP-5024 and DSM40024) were initially tested for pyridomycin production with limited success, likely due to the presence of producing and non-producing populations in the same culture. Pyridomycin (Fig 1A) was, however, readily produced by and purified from *Dactylosporangium fulvum* (NRRL B-16292) with a yield of 20–40 mg/L at a purity >99% and with an NMR spectrum as previously reported (Kinoshita et al, 1989).

### Anti-bacterial properties of pyridomycin

Pyridomycin has been described to act specifically against mycobacteria, with little or no activity against other Gram-positive and Gram-negative species (Maeda et al, 1953). In order to verify its spectrum of activity, the resazurin reduction microplate assay (REMA) was used to determine the minimum inhibitory concentration (MIC) for various bacteria. From Table 1, it can be clearly seen that pyridomycin is effective against all members of the *Mycobacterium* genus tested including *M. tuberculosis* (strain H37Rv, MIC = 0.31–0.63 µg/ml) and *M. smegmatis* (strain mc^2^ 155, MIC = 0.62–1.25 µg/ml). Pyridomycin, however, showed no detectable activity against other bacteria, including the close relative *C. glutamicum* (all MIC > 100 µg/ml). These data therefore agree with earlier observations (Maeda et al, 1953; Maeda, 1957) and suggest that pyridomycin targets a mycobacterial component that is either sufficiently divergent or absent in other genera.

**Table 1:** Bacterial susceptibility to pyridomycin as measured by resazurin reduction microtitre assay

Bacterium	Pyridomycin MIC (µg/ml)
*Mycobacterium tuberculosis*	0.39
*Mycobacterium bovis BCG*	0.39
*Mycobacterium smegmatis*	0.78
*Mycobacterium marinum*	3.13
*Mycobacterium abscessus*	6.25
*Mycobacterium bolletii*	6.25
*Mycobacterium massiliense*	6.25
*Mycobacterium avium*	12.5
*Corynebacterium glutamicum*	>100
*Corynebacterium diphtheriae*	>100
*Micrococcus luteus*	>100
*Listeria monocytogenes*	>100
*Staphylococcus aureus*	>100
*Bacillus subtilis*	>100
*Enterococcus faecalis*	>100
*Escherichia coli*	>100
*Pseudomonas putida*	>100
*Pseudomonas aeruginosa*	>100
*Salmonella typhimurium*	>100
*Candida albicans*	>100

To further understand the properties of pyridomycin against *M. tuberculosis*, its minimum bactericidal concentration (MBC) was determined and its activity against non-replicating and intracellular *M. tuberculosis* measured. MBC data demonstrated that pyridomycin is bactericidal against *M. tuberculosis* H37Rv at concentrations of 0.62–1.25 µg/ml. Evaluation of pyridomycin activity against non-replicating *M. tuberculosis* using the streptomycin-starved 18b (ss18b) model (Sala et al, 2010) revealed that pyridomycin is not effective, thereby implying that it may target a function involved in active growth. Finally, the intracellular killing activity of pyridomycin was assessed *ex vivo* after infection of activated THP1-derived macrophages. The results indicated that, when left untreated for a 7-day period, intracellular *M. tuberculosis* grew by at least 3 logs, whilst exposure to both pyridomycin (10 µg/ml) and rifampicin (1 µg/ml) prevented any multiplication within the macrophages (Fig 1B). Further controls showed that streptomycin (10 µg/ml) had no impact on the growth of intracellular bacteria while isoniazid (1 µg/ml) was able to reduce the intracellular *M. tuberculosis* load by 1 log (Fig 1B). Pyridomycin is therefore clearly able to enter macrophages and arrest bacterial replication.

### Cytotoxicity of pyridomycin on human cell lines

To determine whether pyridomycin is cytotoxic to human cells, the concentration-dependent cytotoxicity of the compound was assessed on two human cell lines. Data indicated that the amount of pyridomycin needed to kill 50% of HepG2 cells (human hepatic cell line) or A549 cells (human lung epithelium cell line) was 100 and 50 µg/ml, respectively. Pyridomycin therefore shows higher selectivity for *M. tuberculosis* compared to the human cells tested (selectivity index >100-fold), in agreement with a previous finding that pyridomycin shows low toxicity in an acute murine model following 800 mg/kg intraperitoneal injection (Maeda et al, 1953).

### Identification of the pyridomycin target

**Figure 2:**
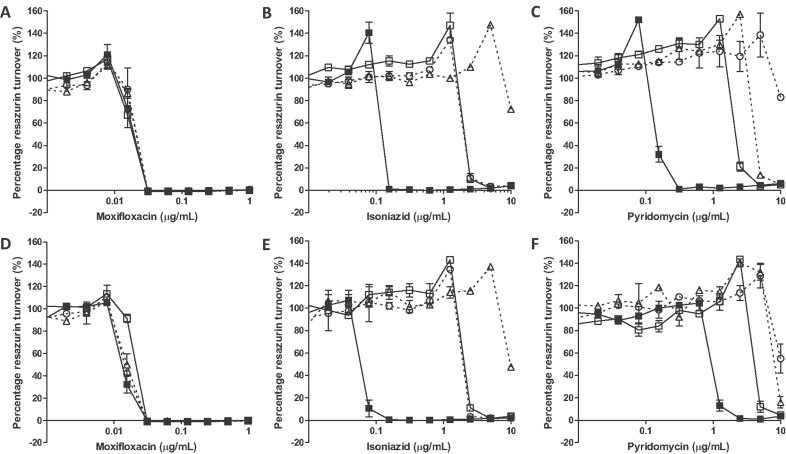
Genetic validation of InhA as the target of pyridomycin **A-C.** The compound susceptibility of wild-type H37Rv transformed with the control vector pMV261 (filled squares), pMVinhA (open squares), pMVinhA (S94A) (open triangle) or pMVinhA (D148G) (open circle) to: (**A**) moxifloxacin, (**B**) isoniazid or (**C**) pyridomycin.**D-F.** The compound susceptibility of pyridomycin-resistant mutant PYR7 transformed with the control vector pMV261 (filled squares), pMVinhA (open squares), pMVinhA (S94A) (open triangle) or pMVinhA (D148G) (open circle) to: (**D**) moxifloxacin, (**E**) isoniazid or (**F**) pyridomycin.

The strategy to identify the target and mechanism of action of pyridomycin was to raise pyridomycin-resistant mutants and to pinpoint the genetic mutations responsible for this phenotype, anticipating that these mutations would be in the gene for the drug target. Resistant mutants of strain H37Rv were selected on solid medium containing pyridomycin at 10× MIC (3 µg/ml) and arose at a frequency of around 10^−6^. Of the 10 colonies selected for further analysis (PYR1 to 10), nine showed no change in the MIC to pyridomycin when re-tested by REMA, whereas mutant PYR7 retained a near 10-fold increase in its resistance level compared to the parent H37Rv (Fig 2). This phenotype was stably maintained and mutant PYR7 remained fully susceptible to isoniazid, moxifloxacin and rifampicin like wild-type H37Rv (Fig 2).

To identify the single nucleotide polymorphisms (SNPs) or insertion/deletions (INDELs) responsible for the pyridomycin resistance, the genomes of both PYR7 and the parental strain were sequenced to near completion by the Illumina protocol. Ninety-eight percent of the reads were successfully mapped to the H37Rv reference genome (Cole et al, 1998) resulting in an average 300-fold coverage. Comparison of the PYR7 and H37Rv assemblies revealed 63 SNPs of which 53 mapped to the repetitive PE and PPE gene families and were therefore discarded. Of the remaining 10 SNPs, nine were synonymous. The only non-synonymous mutation found was an a443g transition in *inhA* resulting in replacement of the aspartic acid at position 148 by a glycine (D148G). This missense mutation was subsequently confirmed by conventional Sanger sequencing. With reference to previously published structures of the NADH-dependent enoyl-ACP reductase InhA, Asp148 was found to be located near the NADH binding pocket (Dessen et al, 1995; Dias et al, 2007; Molle et al, 2010; Oliveira et al, 2006; Rozwarski et al, 1999; Vilcheze et al, 2006).

### Genetic validation of InhA as the target of pyridomycin

To genetically validate InhA as the target of pyridomycin, we evaluated whether over expression of *inhA* caused an increase in resistance to the antibiotic in wild-type *M. tuberculosis*. For this purpose, we transformed strain H37Rv with a plasmid carrying the *inhA* gene under the control of the *hsp60* promoter (pMVinhA; Larsen et al, 2002). In Fig 2, it can be seen that H37Rv::pMVinhA displayed a 15-fold higher MIC for pyridomycin compared to the control strain H37Rv::pMV261 (from 0.31 to 5 µg/ml). When the same experiment was performed in the PYR7 background, no complementation of the resistant phenotype was observed, indicating that the associated mutation was dominant. Similar to the wild-type strain, we noticed a four-fold increase in resistance in PYR7::pMVinhA compared to the empty vector (PYR7::pMV261; from 2.5 to 10 µg/ml; Fig 2). In control experiments, overexpression of *inhA* also led to increased isoniazid resistance in both strains whilst not impacting the MIC of moxifloxacin (Fig 2). Together, these genetic data strongly suggest that InhA is the target of pyridomycin.

To further corroborate that the D148G mutation in *inhA* was indeed responsible for pyridomycin resistance, we overexpressed this allele in H37Rv and compared its effect with that of a well-characterized mutation associated with isoniazid resistance, InhA (S94A) (Vilcheze et al, 2006). Results presented in Fig 2 clearly show that, compared to overexpression of wild-type InhA (pMVinhA), overexpression of InhA (D148G) causes four-fold greater resistance to pyridomycin while overexpression of InhA (S94A) conferred only two-fold resistance. Furthermore, overexpression of InhA (D148G) had no impact on the MIC for isoniazid compared to overexpression of wild type InhA, while, as expected, overexpressing InhA (S94A) increased resistance around four-fold (Fig 2). None of the mutations affected the MIC for moxifloxacin (Fig 2). Collectively, the data prove that the D148G mutation in InhA is responsible for resistance to pyridomycin, whilst not noticeably affecting isoniazid activity.

In addition to isoniazid, InhA is also the target of ethionamide and triclosan. Susceptibility studies using these compounds on H37Rv and PYR7 indicate that both strains are equally sensitive with MICs of 2.0 and 12.5 µg/ml, respectively. This lack of cross-resistance indicates that D148G in InhA is likely to have no impact on the binding to InhA of either triclosan or the active metabolite of ethionamide, the ethionamide-NAD adduct.

### Susceptibility of isoniazid-resistant clinical isolates to pyridomycin

Since our findings indicated that pyridomycin has the same target as isoniazid, we investigated whether isoniazid-resistant clinical isolates of *M. tuberculosis* retained susceptibility to pyridomycin. As isoniazid is a pro-drug, clinically relevant mutations that confer resistance are frequently found in the *katG* gene encoding the catalase–peroxidase required for isoniazid bio-activation or, less commonly, in the promoter region of *inhA*, which increases expression of the protein. Of the eight independent isoniazid-resistant clinical isolates analysed, four had mutations in *katG* (S315T), three in the promoter region of *inhA* [c (−15)t] and one isolate carried both mutations (Table 2). Analysis of the drug susceptibility of these isolates confirmed that all strains carrying the *katG* mutation displayed a high level of resistance to isoniazid (MIC >10 µg/ml) and those isolates carrying only the *inhA* promoter mutation showed intermediate isoniazid resistance (MIC = 1.25 µg/ml) compared to H37Rv (0.16 µg/ml; Table 2). On the contrary, isolates carrying the *katG* mutations showed no resistance to pyridomycin (MIC = 0.3–0.6 µg/ml), while a mutation in the *inhA* promoter resulted in increased pyridomycin resistance (MIC = 2.5–5 µg/ml; Table 2). For all clinical isolates tested, the susceptibility to moxifloxacin was similar to wild-type (MIC = 0.03–0.10 µg/ml). Thus, isoniazid-resistant clinical isolates carrying the *inhA* [*c* (−15)*t*] promoter mutation displayed cross-resistance with pyridomycin, whereas the more common *katG* (*S315T*) isoniazid-resistant mutants retained full sensitivity to the antibiotic.

**Table 2:** Pyridomycin activity against isoniazid-resistant clinical isolates of *M. tuberculosis*

Isolate	KatG genotype^a^	*inhA* promoter genotype^b^	MIC (µg/ml)		
			
			Isoniazid	Pyridomycin	Moxifloxacin
H37Rv	wt	wt	0.16	0.31	0.03
1	S315T	wt	>10	0.63	0.03
2	S315T	wt	>10	0.50	0.05
3	S315T	wt	>10	0.50	0.05
4	S315T	wt	>10	0.31	0.03
5	S315T	c (−15)t	>10	2.5	0.02
6	wt	c (−15)t	1.25	3.75	0.10
7	wt	c (−15)t	1.25	3.75	0.06
8	wt	c (−15)t	1.25	5	0.06

wt, wild-type.

a Numbering refers to the KatG protein sequence.

b Numbering refers to the *inhA* coding sequence, with +1 corresponding to the first base of the ATG start codon.

### Inhibition of mycolic acid synthesis by pyridomycin

**Figure 3:**
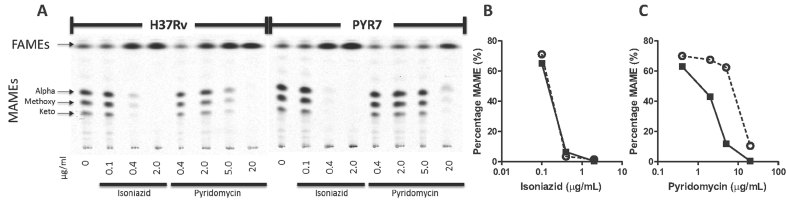
Inhibition of mycolic acid synthesis by pyridomycin The fatty acid methyl ester (FAMEs) and mycolic acid methyl ester (MAMEs) profiles of wild-type H37Rv and pyridomycin-resistant mutant PYR7 were evaluated by thin-layer chromatography. Both strains were treated with different concentrations of isoniazid and pyridomycin for 3 h and labeled with [1,2-^14^C]-acetate. **A.** ^14^C-labeled FAMEs and MAMEs were separated by thin-layer chromatography and detected by autoradiography.**B,C.** Quantification of the MAME band intensity relative to the density of the FAMEs illustrates the inhibition of MAME synthesis by pyridomycin (**B**) and isoniazid (**C**) in H37Rv (black squares) and PYR7 (open circles).

It has been elegantly demonstrated that inhibition of InhA by isoniazid in *M. tuberculosis* leads to the specific depletion of mycolic acids from the bacterial cell wall without affecting fatty acid synthesis (Vilcheze et al, 2006). To show that pyridomycin inhibition of InhA also results in inhibition of mycolic acid synthesis, the mycolic and fatty acid content of *M. tuberculosis* was determined in the presence and absence of pyridomycin by radiometric thin layer chromatography (TLC). We found that pyridomycin caused a concentration-dependent reduction of mycolic acid synthesis (alpha-, methoxy- and keto-mycolic acids) whilst not affecting the fatty acid content (Fig 3). Furthermore, when performing the same experiment on PYR7, over five-fold higher pyridomycin concentrations were needed to inhibit mycolic acid biosynthesis consistent with the resistance level observed. Both H37Rv and PYR7 behaved similarly when the assay was repeated in the presence of isoniazid (Fig 3). Indeed, the latter caused a concentration-dependent decrease in the amount of mycolic acids in H37Rv and was equally effective at inhibiting mycolic acid synthesis in PYR7. Taken together, these data confirm that pyridomycin targets mycolic acid synthesis and demonstrate that the InhA (D148G) enzyme in PYR7 is much less susceptible to pyridomycin inhibition.

### *In vitro* inhibition of InhA by pyridomycin

**Figure 4:**
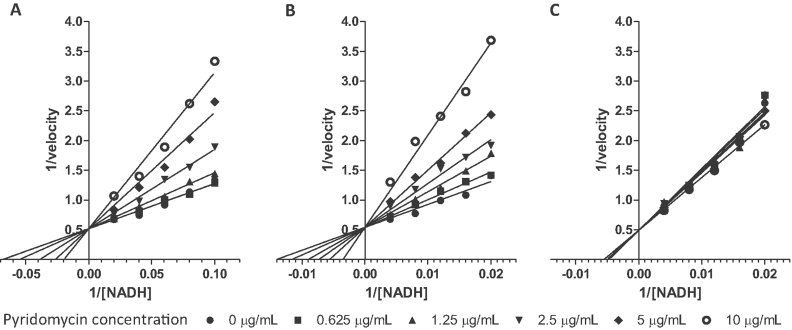
Inhibition of purified InhA by pyridomycin **A-C.** Lineweaver–Burk plot showing the competitive inhibition of wild-type InhA (**A**), InhA (S94A) (**B**) and InhA (D148G) (**C**) by pyridomycin in the presence of NADH.

Inhibition of purified InhA by pyridomycin was studied to investigate if pyridomycin alone can inhibit the enzyme or whether *in vivo* bio-activation by an intracellular process is needed. InhA, InhA (S94A) and InhA (D148G) were successfully expressed and purified. All three enzymes were catalytically active and oxidized NADH in the presence of the substrate 2-trans-octenoyl-CoA (OcCoA). Initial experiments determined the NADH-binding constant (*K*_m_) and confirmed that for InhA (S94A) it was around 6.5 times higher than for wild-type InhA (Table 3) as reported previously (Quemard et al, 1995; Rawat et al, 2003; Vilcheze et al, 2006). Surprisingly, we found that the *K*_m_ of InhA (D148G) for NADH was 14-fold greater than for wild-type InhA (Table 3) suggesting a lower affinity for NADH in the D148G mutant. All the enzymes had a similar *V*_max_ (around 0.52 µmol/min/mg) (Table 3). Enzyme inhibition studies showed that pyridomycin was able to inhibit both wild-type InhA and InhA (S94A) at a similar *K*_*i*_ (6.5 and 4.55 µM, respectively) (Table 3). InhA (D148G) could not be inhibited at all by pyridomycin at concentrations below 18.6 µM. Statistical analysis of inhibition of both wild-type InhA and InhA (S94A) by pyridomycin favours a model of competitive inhibition with NADH as indicated by similar *y*-axis intercepts on Lineweaver–Burk plots (Fig 4). These data prove that pyridomycin is the pharmacophore that inhibits InhA, and this activity is achieved by competitive inhibition of the NADH-binding site. Additionally, these biochemical and enzymological results confirm that InhA (D148G) is more resistant to pyridomycin while InhA (S94A) is as susceptible as the wild-type enzyme.

**Table 3:** *In vitro* kinetic parameters of *M. tuberculosis* InhA and its inhibition by pyridomycin

	Wild-type InhA	InhA (S94A)	InhA (D148G)
NADH *K*_m_ (µM)	13.5 ± 2.3	83.5 ± 9.5	190 ± 16
NADH *V*_max_ (µmol/min/mg)	0.52 ± 0.03	0.50 ± 0.02	0.54 ± 0.3
Pyridomycin *K*_i_ (µM)	6.5 ± 1.2	5.0 ± 0.4	No inhibition at 18.6 µM

### Crystal structures of InhA (D148G), wild-type InhA and InhA (S94A)

To further investigate the mode of binding of pyridomycin to InhA at the atomic level, we crystallized and solved the structures of wild-type InhA and InhA (S94A) mutant in complex with NADH as previously published (Dessen et al, 1995; Dias et al, 2007; Molle et al, 2010; Oliveira et al, 2006; Rozwarski et al, 1999; Vilcheze et al, 2006). In an attempt to obtain crystal structures of InhA in complex with pyridomycin, pre-crystallized InhA:NADH or InhA (S94A):NADH crystals were soaked in a pyridomycin solution. On penetration of pyridomicin, the crystals turned yellow but lost their ability to diffract. This suggests that pyridomycin may induce major conformational changes upon binding to the NADH co-factor pocket of InhA. For control purposes, InhA:NADH crystals were also soaked with triclosan, an inhibitor of the enoyl-ACP substrate binding site of InhA, and the structure successfully solved, thereby ruling out technical issues with soaking (data not shown). As an alternative strategy to define the pyridomycin binding site in InhA attempts were made to co-crystallize InhA or InhA (S94A) in the presence of pyridomycin alone or with the octenoyl CoA substrate; however, despite testing over 1000 conditions, no diffracting crystals have been obtained to date.

**Figure 5:**
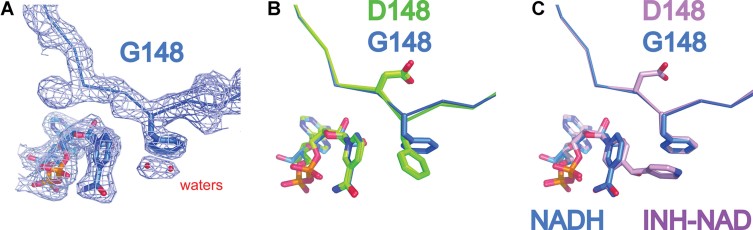
Crystal structures of InhA (D148G) compared to wild-type InhA and InhA (S94A) The 2fo-fc electron density map at 1 sigma of InhA (D148G):NADH structure (at 2.45 Å) with Phe149 and NADH represented as sticks.Superposition of InhA (D148G):NADH (blue) on InhA:NADH and InhA (S94A):NADH (both in green) clearly shows the D148G mutation and the resulting 90° rotation of Phe149.Overlay of InhA (D148G):NADH (Blue) and InhA:NAD-INH (PDB code 1Z1D: Pink) shows that, in this case, Phe149 occupies the same position and explains why InhA (D148G) is still sensitive to inhibition by isoniazid.

The D148G mutation leads to pyridomycin resistance as well as to a decrease in NADH affinity (Table 3). To determine the molecular basis for this resistance, we crystallized InhA (D148G) in the presence or absence of NADH. As with InhA and InhA (S94A), we obtained crystals only in presence of NADH and solved the InhA (D148G):NADH structure to 2.54 Å (Supporting Information Table S3). By comparing the structure of InhA (D148G):NADH with that of wild-type InhA:NADH and InhA (S94A):NADH obtained in this study and elsewhere (Dessen et al, 1995; Dias et al, 2007; Molle et al, 2010; Oliveira et al, 2006; Rozwarski et al, 1999; Vilcheze et al, 2006), we observed only one major effect, namely rotation of the phenylalanine 149 (Phe149) side chain by 90° (Fig 5). Otherwise, the positions of other active site residues as well as the overall structure of the protein were identical (Supporting Information Fig 1).

Interestingly, Phe149 in the InhA (D148G) mutant adopts the same orientation as InhA and InhA (S94A) in complex with INH-NADH (Dias et al, 2007; Rozwarski et al, 1999; Vilcheze et al, 2006). Thus, in the InhA (D148G) mutant, Phe149 is naturally placed for Pi-stacking with the INH moiety and this explains the unchanged MIC observed for isoniazid against wild-type InhA and the InhA (D148G) mutant *in vivo* (Fig 5). Furthermore, rotation of the Phe149 side chain in the InhA (D148G) mutant also results in opening of a water channel allowing the addition of two water molecules in the active site as well as increasing the distance between the nicotinamide ring of NADH and the ring of Phe149, leading to a decrease in NADH affinity as confirmed by kinetic studies (Fig 5). The same structural changes most probably also explain the decreased sensitivity of InhA (D148G) to pyridomycin compared to wild-type as NADH and pyridomycin share a similar binding pocket.

## DISCUSSION

To inhibit or kill competing organisms, numerous microbes produce and secrete natural products with antibiotic activity. This rich source of chemically diverse compounds was successfully exploited for the production of many anti-microbial and anti-cancer drugs in the first decades of antibiotic development but was then abandoned (Fischbach & Walsh, 2009). Since the selective pressure imparted by some antibiotics with broad-spectrum activity may have led to naturally occurring resistance in other bacteria that share the same ecological niche, many potentially bio-active molecules may have been overlooked (Smith et al, 2010). The ecological and evolutionary forces that have shaped natural products, particularly the selection and inhibition of vulnerable targets, remain unknown but understanding this relationship is important for drug discovery.

The treatment of tuberculosis is increasingly menaced by the emergence of drug-resistant strains of *M. tuberculosis* and this has led to renewed interest in finding new bactericidal inhibitors. As in other anti-infective areas, target-based screens have been unsuccessful prompting investigators to adopt whole cell screening once more (Cole & Riccardi, 2011; Payne et al, 2007). Consequently, we are reinvestigating diverse natural products for hit generation as these compounds have been optimized by evolution as antibacterial agents. In this study, we have used pyridomycin, a natural product with anti-mycobacterial activity, to identify the intracellular target and characterized its mechanism of action. Pyridomycin was first described in 1953 (Maeda et al, 1953), shortly after the introduction of isoniazid into clinical practice, but was then apparently neglected. In a remarkable coincidence, we show that, like isoniazid, pyridomycin directly targets the NADH-dependent enoyl (ACP)-reductase InhA and causes inhibition by competing for the NADH-binding pocket.

InhA (also known as FabI) is an essential component of the type II fatty acid synthase system (FasII) involved in fatty acid elongation and is required for mycolate production in *M. tuberculosis*. The FasII system is highly conserved in bacteria but absent from humans making it an attractive drug target (Heath et al, 2002; McMurry et al, 1998; Vilcheze et al, 2006, 2011). Although isoniazid is certainly the most effective known inhibitor of InhA in *M. tuberculosis*, it is a pro-drug requiring activation by the KatG catalase–peroxidase to form an adduct with NAD. Clinically significant resistance to isoniazid is mainly attributed to loss or alteration of KatG activity. The INH-NAD adduct acts as a slow, tightly binding competitive inhibitor of the NADH-binding site of InhA (Rawat et al, 2003). Interestingly, mycobacterial InhA is also targeted by the small molecules ethionamide (Morlock et al, 2003) (also a pro-drug) and triclosan (Parikh et al, 2000) whilst in other bacteria, FabI is inhibited by the natural products vinaxanthone and cephalochromin (Zhang & Rock, 2008). Other natural products with broad-spectrum activity, such as thiolactomycin, cerulenin and platensimycin, have been shown to target different components of the FasII system in other bacteria (Zhang & Rock, 2008). Our results suggest that pyridomycin is the most potent natural product to inhibit FasII specifically in *M. tuberculosis* and resistance arises due to remodelling of the NAD-binding site in InhA, notably through mutation of D148G.

Pharmacologically validated drug targets are scarce in *M. tuberculosis* with InhA being among the best (Lamichhane, 2011). For this reason, several attempts have been made to develop heterocyclic inhibitors that differ from isoniazid and ethionamide in their structure and activity. Examples include heterocyclic boron containing compounds (diazaborine) that react with NAD^+^ ribose to form diazyborine-NAD adducts that inhibit InhA similarly to INH-NAD (Baldock et al, 1996). Triclosan analogues (Freundlich et al, 2009; Vilcheze et al, 2011) and di-phenyl ether compounds (Sullivan et al, 2006) have been shown to inhibit InhA with nanomalar *K*_i_ and micromolar MICs and are both promising candidates as anti-tuberculosis compounds. Further screening studies for *Escherichia coli* FabI inhibitors have also revealed effective novel structures, many of which however do not have good MIC against *M. tuberculosis* (Lu & Tonge, 2008; Payne et al, 2002).

It is noteworthy that, while the FasII system is present in most bacteria, pyridomycin is a specific inhibitor of mycobacterial species (Table 1). Amongst the mycobacteria tested here, the InhA proteins share a high level of sequence identity (77%) and both Asp148 and Phe149 (as well as other active site residues) are strictly conserved, which may explain why all mycobacteria were susceptible to the antibiotic. Additionally, other pathogenic mycobacteria such as *M. leprae* and *M. ulcerans* also share near identical InhA proteins (91 and 93% identity to the *M. tuberculosis* ortholog, respectively) and are expected to be susceptible to pyridomycin (Supporting Information Fig 1). The level of sequence identity between *M. tuberculosis* InhA and FabI from the different Gram-positive and Gram-negative bacteria tested here is considerably lower, ranging from 27 to 33%. Neither Asp148 nor Phe149 are conserved in these enzymes, which probably accounts for their pyridomycin resistance. It is possible that through cohabitating with producers of pyridomycin, or a related natural product, the ancestors of these bacteria may have acquired resistance to the antibiotic as has been proposed for arylomycin, a natural product that inhibits signal peptidase I (Smith et al, 2010).

The increasing emergence of isoniazid resistance in clinical isolates of *M. tuberculosis* is an important problem for tuberculosis therapy and seriously compromises the effectiveness of current treatment. In 50–95% of the cases, resistance to isoniazid is caused by mutations in *katG* (Zhang & Yew, 2009). Low-level resistance to isoniazid is also associated with upregulation of *inhA* but mutations in the *inhA* gene itself are much less common (8–43%) (Zhang & Yew, 2009). Our data clearly show that pyridomycin does not require activation. This is of particular significance because it means that pyridomycin can effectively kill isoniazid-resistant *M. tuberculosis* carrying *katG* mutations as demonstrated by our susceptibility testing of isoniazid-resistant clinical isolates.

The pyridomycin resistant strain PYR7, that carries the D148G mutation in InhA, is not cross-resistant to isoniazid and ethionamide while, conversely, the S94A variant that displays isoniazid resistance remains susceptible to pyridomycin (Fig 2). This suggests that, while both pharmacophores are competitive inhibitors of NADH-binding, they bind to the pocket in different ways. Additionally, the lack of cross resistance with triclosan, the scaffold for other InhA inhibitor programs, is promising as it demonstrates that there are multiple ways of inhibiting the same protein without cross-resistance occurring. These are important findings for rational drug design and could lead to the development of pyridomycin derivatives that kill multiple mycobacteria unlike isoniazid, which is effective solely against *M. tuberculosis*.

## MATERIALS AND METHODS

### General information

Bacterial strains, culture conditions, pyridomycin production and purification, expression and purification of proteins and details of materials are described in Supporting Information Materials and Methods.

### Determination of pyridomycin MIC

The drug susceptibility of all bacteria was determined using the resazurin microtitre assay (REMA; Palomino et al, 2002). Briefly, log-phase bacteria were diluted to an OD_600_ of 0.0001, and grown in a 96-well plate in the presence of serial compound dilutions. After 10 generations (7 days for *M. tuberculosis*) bacterial viability was determined using 10 µl of resazurin (0.025% w/v), and calculated as a percentage of resazurin turnover in the absence of compound. Methodology for determining the minimum bactericidal activity (MBC) and intracellular activity are described in the Supporting Information Materials and Methods.

### Isolation and characterization of pyridomycin-resistant H37Rv clones

Pyridomycin-resistant H37Rv mutants were isolated by plating 10^9^ CFU on solid 7H10 medium containing 3 µg/ml of pyridomycin (10 × MIC). Following 4 weeks of incubation (37 °C), colonies were picked and grown in 7H9 medium without pyridomycin. Colonies were then retested for susceptibility to pyridomycin, moxifloxicin, isoniazid and rifampicin. Pyridomycin-resistant clone PYR7 was selected for whole genome sequencing using Illumina technology and reads aligned to the genome sequence of the parent H37Rv genome to identify SNPs (protocols described in Supporting Information Materials and Methods). The SNP in *inhA* was validated by conventional Sanger sequencing using an ABI3130XL genetic analyser (Applied Biosystems).

### Determination of MIC on isoniazid-resistant clinical isolates

Nine isoniazid-resistant clinical isolates that were ‘genotyped’ using the Line probe assay (1st line drugs, GenoType MTBDRplus) were kindly supplied by the Centre Hospitalier Universitaire Vaudois (CHUV) and the Hôpitaux Universitaires de Genève (HUG). Isolates were grown to mid log phase in liquid medium and tested for their pyridomycin, isoniazid and moxifloxacin susceptibility by REMA.

### Over-expression of *inhA* in H37Rv and PYR7

To genetically validate that InhA was the target of pyridomycin, the impact of over-expressing either wild-type InhA or the mutant forms in strains H37Rv and PYR7 was determined. Briefly, Quick change site-directed mutagenesis was used with pMVInhA [where *inhA* is expressed from the *hsp60* promoter in pMV261 (Larsen et al, 2002)] to introduce t280g or a443g mutations thus generating pMVInhA (S94A) and pMVInhA (D148G), respectively (primers in Supporting Information Table 1). Plasmids pMVInhA, pMVInhA (S94A) and pMVInhA (D148G) were transformed into H37Rv and PYR7. Transformants were selected using kanamycin, then verified by colony PCR for the presence of the kanamycin-resistance cassette before determining their MIC for pyridomycin, isoniazid and moxifloxacin by REMA.

### Inhibition of mycolic acid synthesis by pyridomycin

The inhibition of mycolic acid production by pyridomycin and isoniazid was determined as previously described (Vilcheze et al, 2006). Briefly, early log-phase cultures of H37Rv and PYR7 (OD_600_ = 0.3, 4 ml) were treated for 3 h with isoniazid (0.1, 0.4 and 2 µg/ml) or pyridomycin (0.4, 2, 5, and 20 µg/ml) prior to labelling for 20 h with [1,2-^14^C] acetate (1 µCi/ml; Perkin Elmer). Bacteria were harvested and washed with double-distilled H_2_O to remove excess [1,2-^14^C]-acetate. The bacterial pellet was then treated with 2 ml of 20% tetrabutyl ammonium hydroxide overnight at 100 °C to extract the mycolic acids from the cell wall. Mycolic acids were subsequently methylated and extracted by incubation with an equal volume of methylene chloride and 100 µl of methyl iodide for 1 h at room temperature with mixing. The organic phase containing the mycolic acid methyl esters (MAMEs) was washed once with 3N HCl and once with H_2_O, dried under nitrogen and resuspended in a smaller volume of methylene chloride. Radiolabelled MAMEs were analysed by TLC. For each condition 10,000 dpm were loaded on a HPTLC Silica Gel 60 F254 plate and run three times with 95:5 v/v hexane/ethyl acetate. The MAMEs were visualized using a Typhoon™ scanner (GE Healthcare) and quantified with the software ImageQuant™ (GE Healthcare).

**The paper explained**

PROBLEM:

Even today, infection with *Mycobacterium tuberculosis* accounts for up to two million deaths annually. The effectiveness of current anti-tuberculosis drugs to combat these infections is increasingly compromised by the escalating prevalence of multi- and extensively drug-resistant tuberculosis. For these cases, the most effective anti-tubercular compounds such as isoniazid and rifampicin are no longer effective and this can result in mortality rates approaching 100% for patients with extensively drug-resistant tuberculosis. For these reasons, it is imperative to ensure that the pipeline of drug candidates to treat tuberculosis is well filled.

RESULTS:

We show here that the natural product pyridomycin is a very selective bactericidal compound against mycobacteria including *Mycobacterium tuberculosis*, the causative bacterium of tuberculosis in humans. By selecting and isolating *M. tuberculosis* mutants resistant to pyridomycin and sequencing their genome, we found that a single mutation in a gene named *inhA* is responsible for the resistance. InhA is already the target of the current frontline anti-tuberculosis agent isoniazid. However, most interestingly, no cross resistance was observed between pyridomycin and isoniazid, both in laboratory strains containing mutations in InhA or in the most frequently encountered isoniazid-resistant clinical isolates that contain mutations in *katG* (a gene required to activate isoniazid). We then present detailed genetic and biochemical studies to confirm that pyridomycin itself inhibits InhA and that in live bacteria, this inhibition leads to the depletion of mycolic acids, an essential cell wall component. Finally, studies of the crystal structure of the InhA protein and the pyridomycin-resistant form give valuable insight into the binding pocket of pyridomycin.

IMPACT:

Inhibition of InhA is one of the most effective means of killing *Mycobacterium tuberculosis*, and this is the mechanism behind one of the most potent anti-tubercular agents currently used: isoniazid. The increasing emergence of multi- and extensively drug-resistant tuberculosis (both of which are resistant to isoniazid) means that for these cases, this target can no longer be effectively inhibited by current therapy. Our finding that pyridomycin kills *M. tuberculosis* by inhibiting InhA (even in isoniazid-resistant clinical isolates) provides a promising basis for the development of pyridomycin or a related agent for the treatment of isoniazid-resistant tuberculosis.

### Steady state kinetics and inhibition of InhA

Inhibition of InhA activity was investigated using InhA, InhA (S94A) and InhA (D148G) as described previously (Dessen et al, 1995; Quemard et al, 1995). Briefly, kinetic parameters were determined by following NADH oxidation at 340 nm using a TECAN FL200 spectrophotometer. All reactions were performed at 25 °C with 100 nM InhA (or mutants) in 30 mM PIPES (pH 6.8), 150 mM NaCl and 10% glycerol. After addition of variable concentrations of NADH, reactions were initiated by adding 2-trans-octenoyl-CoA (synthesized as described in Supporting Information Materials and Methods) to a final concentration of 250 µM. Steady state *K*_m_ for NADH was determined by measuring enzyme kinetics at different NADH concentrations (0–800 µM). NADH *K*_m_ and pyridomycin *K*_i_ were determined by measuring enzyme kinetics with both different NADH (wild type InhA – 10, 12.5, 16.7, 25 and 50 µM, for both InhA (S94A) and InhA (D148G) −50, 62.5, 83.3, 125, 250 µM NAHD) and different pyridomycin concentrations (0, 0.625, 1.25, 2.5, 5 and 10 µg/ml). Kinetic parameters were analysed and calculated using GraphPad Prism 5.

### Crystallization, data collection and structure determination

Crystals of the InhA:NADH complex, InhA (D148G):NADH complex and InhA (S94A):NADH complex were obtained by vapour diffusion at 18°C by equilibrating 2 µl hanging drops containing a 1:1 mixture of approximately 10 mg/ml protein incubated with 5 mM NADH and crystallization buffer (10% v/v MPD and 0.1 M TRIS, pH 8.0 for InhA:NADH complex; 8% v/v MPD and 0.1 M Bicine, pH 9.0 for InhA (D148G):NADH complex; and 8% v/v MPD, 50 mM Sodium Citrate, pH 6.5 and 0.1 M Hepes, pH 7.5 for InhA (S94A):NADH complex) over a 500 µl reservoir of the same crystallization solution. Crystals were stabilized by soaking briefly in a cryoprotectant solution (25% glycerol w/v in crystallization buffer) and flash frozen in liquid nitrogen before data collection. Diffraction data were collected on X06DA of the Swiss Light Source (SLS, PSI, Villigen, Switzerland) and on ID29 at the European Synchrotron Radiation Facility (ESRF, Grenoble, France). Data were indexed, integrated and scaled with XDS (Kabsch, 2010). Both wild-type InhA and its mutants crystallized in the P6222 space group with unit cell dimensions of approximately *a* = *b* = 98 Å, *c* = 139 Å, *α* = *β* = 90° and *γ* = 120°, with one molecule per asymmetric unit (Supporting Information Table 2). Phase determinations were carried out by molecular replacement using Phaser (McCoy et al, 2007), part of the CCP4 Suite, using as a search model the published structure of the InhA:NADH complex from *M. tuberculosis* (PDB code 3OEW). The initial molecular replacement models were manually adjusted in COOT, part of the CCP4 Suite (Winn et al, 2011) and refined with REFMAC5 (Murshudov et al, 1997). The refined structures were evaluated with PROCHECK (Laskowski et al, 1996). Structure figures were prepared with PyMOL (Molecular Graphics System, Version 1.5.0.1 Schrödinger, LLC). All crystallographic statistics are listed in Supporting Information Table 2. Coordinates and structure factors for the above complexes structures have been deposited in the Protein Data Bank (PDB accession codes 4DRE, 4DTI and 4DQU, respectively).

## Supplementary material

Detailed facts of importance to specialist readers are published as ”Supporting Information”. Such documents are peer-reviewed, but not copy-edited or typeset. They are made available as submitted by the authors.
