# Supplementary material for: Towards a new tuberculosis drug: pyridomycin – nature's isoniazid
Source: EMBO Mol Med. 2012 Sep 17;4(10):1032–42. doi: 10.1002/emmm.201201689 (PMC3491834; doi:10.1002/emmm.201201689)
Supplement: Supplementary file 1 [file emmm0004-1032-SD1.pdf]

EMBO
Molecular Medicine

Manuscript EMM-2012-01689

Towards a new tuberculosis drug: Pyridomycin - Nature's Isoniazid

Ruben C. Hartkoorn, Claudia Sala1, João Neres, Florence Pojer, Sophie J. Magnet, Raju Mukherjee, Swapna Uplekar, Stefanie Boy-Röttger, Karl-Heinz Altmann and Stewart T. Cole

Corresponding author: Stewart T. Cole, EPFL

Review timeline:

Submission date:	29 June 2012
Editorial Decision:	19 July 2012
Revision received:	26 July 2012
Accepted:	30 July 2012

Transaction Report:

(Note: With the exception of the correction of typographical or spelling errors that could be a source of ambiguity, letters and reports are not edited. The original formatting of letters and referee reports may not be reflected in this compilation.)

1st Editorial Decision 19 July 2012

Thank you for the submission of your manuscript to EMBO Molecular Medicine. We have now received the enclosed three reports from the referees that were asked to assess it. As you will see the reviewers are supportive and I am pleased to inform you that we will be able to accept your manuscript pending the following final amendments:

- Please take the comments of Referee #3 into account to revise your manuscript.

Please submit your revised manuscript within two weeks. Please see below for further important information regarding your paper processing.

I look forward to reading a new revised version of your manuscript as soon as possible.

Yours sincerely,

Editor
EMBO Molecular Medicine

***** Reviewer's comments *****

Referee #1 (Comments on Novelty/Model System):

The tuberculosis-specific natural product pyridomycin was discovered in 1953 but not commercialized. In this study the authors convincingly demonstrate that InhA, the enzyme that is targeted by the front line drug isoniazid is the target of pyrdiomycin, but at a distinct subsite.

Referee #1 (Other Remarks):

The authors take up the old natural product pyridomycin (1953) which has narrow specificity for Mtb. Through a range of modern methods from genome sequencing of resistant mutants, to overproduction efforts of InhA wild type and inh-resistant alleles, and biochemistry of purified InhA, the authors conclusively demonstrate a separate NADH subsite is the p[yridomycin target. This is a model paper for how to identify the target of an Mtb-active molecule. And maybe pyridiomycin and isoniazid should be used in combination. Publish as is, without miodification

Referee #2 (Comments on Novelty/Model System):

This is an excellent manuscript with high novelty and impact. The authors explicitly demonstrated the target of the antibiotic:Pyridomycin through a logically sound design and procedure. It should be accepted without revision except minor changes.

Referee #2 (Other Remarks):

This is an excellent work. All designs and procedures look good to me.
1. The only regret is that lack of complex structure of the enzyme and Pyridomycin. Based on the information the authors supplied, it seems extremely hard to obtain high quality crystals of the complex of the enzyme and Pyridomycin. One suggestion is that use the microcrystals of the enzyme without NADH as seeds and droping these seeds in solution of the complex to see if you have a better luck.

2. It is suspicious to put two water molecules close to the F149 ring, the low resolution is not good enough to tell if it is other small molecule.

3. It may be a good idea to model the Pyridomycin into the enzyme active center to explain the potential competitive feature of the antibiotic.

Referee #3:

The authors describe their investigation of a previously ignored anti-mycobacterial compound very lucidly. They used well-established molecular genetic, biochemical, protein structural and enzymatic techniques to document its inhibitory activity, identify the enzyme it targets and the mechanism by which it acts on that enzyme. The drug does, as they claim, potentially provide a backup for isoniazid - one of the most important front-line anti-tuberculosis drugs. The results of the present study appear sufficiently encouraging that it would be justified to carry out animal studies and, if they are favourable, a phase one trial in humans; although this future work is very clearly well beyond the scope of the present study. The authors should address the following minor points:

1. Line 28. It is an exaggeration to claim that isoniazid is "now of limited use." when it remains a very widely used front-line drug that in most cases is part of effective therapy that leads to tuberculosis cure.

2. Lines 114-119. The statements in this section about the results in Fig 1b are correct but because they are not all described in the same way I had to read them several times to be convinced of that. I am sure the authors could rewrite it in a clearer way.

3. Lines 121-129. These results should be put in context here or in the Discussion by comparison to equivalent results with other drugs such as INH and rifampicin.

4. Line 529. Similar exaggeration to point 1; the implication from their statement is that INH should no longer be used, which is wrong. Their statement is only true for a minority of cases - the drug-resistant ones - and the authors do not make this clear.

5. Supplementary figure 2. It would be better if the order of alignments was in order of degree of

similarity. So for example Bovis and not Leprae should be immediately underneath Tuberculosis as the sequences of Tuberculosis and Bovis are identical.

1st Revision - Authors' Response 26 July 2012

Response to referees

- Editors comment: Please take the comments of Referee #3 into account to revise your manuscript.

Referee #1 (Comments on Novelty/Model System):

The tuberculosis-specific natural product pyridomycin was discovered in 1953 but not commercialized. In this study the authors convincingly demonstrate that InhA, the enzyme that is targeted by the front line drug isoniazid is the target of pyridomycin, but at a distinct subsite.

The authors take up the old natural product pyridomycin (1953) which has narrow specificity for Mtb. Through a range of modern methods from genome sequencing of resistant mutants, to overproduction efforts of InhA wild type and inh-resistant alleles, and biochemistry of purified InhA, the authors conclusively demonstrate a separate NADH subsite is the pyridomycin target. This is a model paper for how to identify the target of an Mtb-active molecule. And maybe pyridomycin and isoniazid should be used in combination. Publish as is, without modification

Referee #2 (Comments on Novelty/Model System):

This is an excellent manuscript with high novelty and impact. The authors explicitly demonstrated the target of the antibiotic: Pyridomycin through a logically sound design and procedure. It should be accepted without revision except minor changes.

This is an excellent work. All designs and procedures look good to me.

1. The only regret is that lack of complex structure of the enzyme and Pyridomycin. Based on the information the authors supplied, it seems extremely hard to obtain high quality crystals of the complex of the enzyme and Pyridomycin. One suggestion is that use the microcrystals of the enzyme without NADH as seeds and dropping these seeds in solution of the complex to see if you have a better luck.

> *This suggested approach to co-crystallise InhA with pyridomycin using "seeds" has already been attempted but they have unfortunately not lead to the improvement of crystals.*

2. It is suspicious to put two water molecules close to the F149 ring; the low resolution is not good enough to tell if it is other small molecule.

> *We tried to fit alternative molecules, such as the ones present in the crystallization condition and in the cryoprotectant solution. None of them fitted as good as water molecules. We also want to point out that the B factor of those 2 waters are very similar to the amino acids around them. Moreover, we think that the resolution of the structure is high enough to model water molecules in an accurate manner.*

3. It may be a good idea to model the Pyridomycin into the enzyme active centre to explain the potential competitive feature of the antibiotic.

This approach has also already been attempted using crystal structures of InhA, with NADH removed. The removal of NADH however leaves a very big binding pocket into which pyridomycin can dock in numerous ways, therefore making it difficult to have confidence in any particular docking form. Additionally, InhA is a dynamic protein and currently we are not sure to which particular conformation of InhA, pyridomycin binds. Altogether we therefore did not have sufficient faith in the docking studies to present it in this manuscript.

Referee #3:

The authors describe their investigation of a previously ignored anti-mycobacterial compound very lucidly. They used well-established molecular genetics, biochemical, protein structural and enzymatic techniques to document its inhibitory activity identify the enzyme it targets and the mechanism by which it acts on that enzyme. The drug does, as they claim, potentially provide a backup for isoniazid - one of the most important front-line anti-tuberculosis drugs. The results of the present study appear sufficiently encouraging that it would be justified to carry out animal studies and, if they are favourable, a phase one trial in humans; although this future work is very clearly well beyond the scope of the present study. The authors should address the following minor points:

1. Line 28. It is an exaggeration to claim that isoniazid is "now of limited use." when it remains a very widely used front-line drug that in most cases is part of effective therapy that leads to tuberculosis cure.

The authors agree with this comment and the phrase "now of limited use" has been removed. It now reads:

"Tuberculosis, a global threat to public health, is becoming untreatable due to widespread drug resistance to frontline drugs such as the InhA-inhibitor isoniazid."

2. Lines 114-119. The statements in this section about the results in Fig 1b are correct but because they are not all described in the same way I had to read them several times to be convinced of that. I am sure the authors could rewrite it in a clearer way.

This section has been rephrased to clarify the statement. The paragraph now reads:

"The results indicated that when left untreated for a 7-day period intracellular M. tuberculosis grew by at least 3 logs, whilst exposure to both pyridomycin (10 µg/ml) and rifampicin (1 µg/ml) prevented any multiplication within the macrophages (Fig 1B). Further controls showed that streptomycin (10 µg/ml) had no impact on the growth of intracellular bacteria while isoniazid (1 µg/ml) was able to reduce the intracellular M.tuberculosis load by 1-log (Fig 1B)."

3. Lines 121-129. These results should be put in context here or in the Discussion by comparison to equivalent results with other drugs such as INH and rifampicin.

This section concerns the relative lack of cytotoxicity seen with pyridomycin. These experiments were performed to show that in vitro pyridomycin seems relatively non-toxic, but the authors believe that it is not necessary to compare this with existing anti-tuberculosis compounds that of course also have very low cytotoxicity. The authors would prefer to keep this section as originally submitted.

4. Line 529. Similar exaggeration to point 1; the implication from their statement is that INH should no longer be used, which is wrong. Their statement is only true for a minority of cases - the drug-resistant ones - and the authors do not make this clear.

> *This section in the impact section of "the paper explained" has been rephrased to clarify that we don't think that "isoniazid should no longer be used". It now reads:*
>
> *"Inhibition of InhA is one of the most effective means of killing Mycobacterium tuberculosis, and this is the mechanism behind one of the most potent anti-tubercular agents currently used: isoniazid. The increasing emergence of multi- and extensively-drug resistant tuberculosis (both of which are resistant to isoniazid), means that for these cases this target can no longer be effectively inhibited by current therapy."*

5. Supplementary figure 2. It would be better if the order of alignments was in order of degree of similarity. So for example Bovis and not Leprae should be immediately underneath Tuberculosis as the sequences of Tuberculosis and Bovis are identical.

> *The order of the protein alignments has been changed to have M.bovis after M.tuberculosis.*
