# Supplementary material for: Towards a new tuberculosis drug: pyridomycin – nature's isoniazid
Source: EMBO Mol Med. 2012 Sep 17;4(10):1032–42. doi: 10.1002/emmm.201201689 (PMC3491834; doi:10.1002/emmm.201201689)
Supplement: Supplementary file 2 [file emmm0004-1032-SD2.pdf]

Supporting Information For:

Towards a new tuberculosis drug: Pyridomycin - Nature's Isoniazid

Ruben C. Hartkoorn, Claudia Sala, Joao Neres, Florence Pojer, Sophie J. Magnet, Raju Mukherjee, Swapna Uplekar, Stefanie Boy-Röttger, Karl-Heinz Altmann & Stewart T. Cole

Table of contents

Supplementary Methods **S2**

Bacterial strains, cell lines, culture conditions and chemicals S2

Purification of pyridomycin S2

Determination of minimum bactericidal activity (MBC) S3

Infection of THP1-derived macrophages and evaluation of pyridomycin activity ex vivo S3

Pyridomycin cytotoxicity S4

Genomic DNA preparation for Illumina sequencing S4

Library preparation and high-throughput sequencing with Illumina-Solexa platform S5

Alignment of Illumina sequence reads and identification of single nucleotide polymorphisms S5

Expression and purification of InhA S6

Synthesis of 2-trans-octenoyl CoA S6

Preparative HPLC: S7

Supplementary Figures **S8**

Supplementary Figure 1 S8

Supplementary Figure 2 S9

Supplementary tables **S10**

Supplementary table 1 S10

Supplementary table 2 S10

References **S11**

Supplementary Methods

Bacterial strains, cell lines, culture conditions and chemicals

Mycobacterium tuberculosis, Mycobacterium abscessus, Mycobacterium avium, Mycobacterium bolletii, Mycobacterium bovis BCG, Mycobacterium marinum, Mycobacterium massiliense and Mycobacterium smegmatis were all routinely grown in Middlebrook 7H9 broth (Difco) supplemented with 0.2% glycerol, 0.05% Tween 80 and 10% albumin-dextrose-catalase (ADC) or on Middlebrook 7H11 plates supplemented with 0.5% glycerol and 10% oleic acid-albumin-dextrose-catalase (OADC). Bacillus subtilis, Candida albicans, Corynebacterium diphtheriae, Corynebacterium glutamicum, Escherichia coli, Micrococcus luteus, Pseudomonas putida, Salmonella typhimurium, Staphylococcus aureus were all grown in LB broth. Enterococcus faecalis, Listeria monocytogenes and Pseudomonas aeruginosa were grown in brain heart infusion (BHI) broth. Human hepatic cell line HepG2 was obtained from ATCC (HB8065) and the lung epithelial cell line A549 was obtained from European Collection of Cell Cultures (ECACC: 86012804). Both cell lines were routinely grown in DMEM (Invitrogen) supplemented with 10% fetal bovine serum (FBS) (Invitrogen). All chemicals were purchased from Sigma-Aldrich, unless stated otherwise.

Purification of pyridomycin

Pyridomycin was produced by and extracted from Dactylosporangium fulvum (NRRL-B-16292), obtained from Agricultural Research Service (ARS). Briefly D. fulvum was grown in GYM media (0.4 % (w/v) glucose, 0.4 % (w/v) yeast extract and 1 % (w/v) malt extract, pH 7.2) for 10 days (30°C, 200 rpm). Bacteria were pelleted (6000 rpm, 30 min) and liquid-liquid extraction was performed on the supernatant with ethyl acetate (3 x 0.5 L of ethyl acetate

per L of culture). The organic layer was washed with brine and the solvent removed under reduced pressure. The residue was purified by chromatography on a Varian 971-FP chromatography system, using pre-packed Luknova silica-gel columns and a gradient of dichloromethane/methanol (0-10%). Further purification was then performed by preparative HLPC-MS using Method 1 (RT=6.4 min). Fractions containing pyridomycin were combined and lyophilized. Identity was confirmed by mass spectrometry and 1H-NMR in comparison to literature data (Kinoshita et al, 1989).

Determination of minimum bactericidal activity (MBC)

The MBC is defined as the compound concentration at which the number of bacteria has decreased by 2 logs compared to the start of compound exposure. The MBC was determined by setting up 2 duplicate microtritre plates (as was performed for MIC determination) with a starting bacterial concentration of 5000 CFU/well. At day 0, 50 µL of a 1 in 100 dilution (2 log decrease) of the bacterial culture was plated on Middlebrook 7H11 solid media (supplemented with 0.5% glycerol and 10% OADC). Following 7 days, one of the microtitre plates was processed to determine the MIC of the compound. On day 11, 50 µL of the 5 wells above the determined MIC were plated on Middlebrook 7H11 solid media (supplemented with 0.5% glycerol and 10 % OADC). The lowest concentration where the CFU count was below 10-2 dilution of the day 0 culture was considered as the MBC.

Infection of THP1-derived macrophages and evaluation of pyridomycin activity ex vivo

THP1 cells grown in RPMI medium with 10% FBS (fetal bovine serum) were seeded at 5x105 cells/well in a 24-well plate and allowed to differentiate into adherent macrophages by activating them with 100 nM PMA (phorbol myristate acetate). Following 3 days, adherent

cells were infected with M. tuberculosis Erdman at an MOI (multiplicity of infection) of 1:1 and incubated at 37°C under 5% CO_2. Four hours post-infection, extracellular bacteria were removed by washing with warm PBS and cells were incubated in RPMI with 10% FBS and 100 nM PMA and treated with INH (isoniazid, 1 µg/ml) or RIF (rifampicin, 1 µg/ml) or STR (streptomycin, 10 µg/ml) or PYR (pyridomycin, 10 µg/ml) in duplicate. An untreated sample (in duplicate) served as negative control. At DAY 7 post-infection, cells were washed with warm PBS, lysed with 0.5% Triton X-100 and serial dilutions were plated on 7H10 plates for enumeration of CFU (colony forming units).

Pyridomycin cytotoxicity

Pyridomycin cytotoxicity was measured against HepG2 and A549 cell lines in a 96-well plate. 4000 cells in DMEM without phenol red (supplemented with 10% FBS) were seeded in each well and grown in the presence of serial dilutions of pyridomycin (100 - 0.2 µg/mL). Following 3 days culture (37°C, 5% CO_2), cell line viability was determined by REMA (10 µL of 0.025% resazurin). Cytotoxicity was expressed as the concentration at which there was 50% resazurin turnover (TD_{50}) compared to the no drug controls.

Genomic DNA preparation for Illumina sequencing

M. tuberculosis H37Rv wild type and the pyridomycin resistant clone (PYR7) were grown in 7H9 complete medium to OD_{600} 0.8. Ten milliliters of culture were collected by centrifugation, cells resuspended in 250 µl SET (25% sucrose, 50 mM EDTA, 50 mM Tris HCl pH 8) and added with 50 ml of 20 µg/ml lysozyme. After overnight incubation at 37°C, the suspension was treated first for 30 minutes at 37°C with 5 µl of 10 mg/ml RNAse A and then for 2 hours at 55°C with 250 µl Proteinase K solution (400 mg/ml Proteinase K, 100 mM Tris

HCl pH8, 0.5% SDS). DNA was extracted once with phenol-chloroform-isoamyl alcohol (25:24:1), once with chloroform-isoamyl alcohol (24:1), precipitated in ethanol, air-dried and resuspended in TE buffer. Amount and purity of DNA were checked by Nanodrop, integrity was verified on 0.6% agarose gel.

Library preparation and high-throughput sequencing with Illumina-Solexa platform

Genomic DNA fragment sequencing libraries were prepared using the TruSeq DNA Sample Prep Kit (Illumina; San Diego, California, USA; Catalog Number FC-121-1001) according to the protocol supplied with the reagents and using 1 μg of genomic DNA purified as described above. The resulting genomic DNA fragment library was loaded into one channel of a single read v4 flowcell (Catalog Number GD-300-2001) and sequenced on the Illumina Genome Analyzer IIx using the 36 Cycle TruSeq SBS Kit v5 (Catalog number FC-104-5020). Data were processed using the Illumina Pipeline Software package v1.7.

Alignment of Illumina sequence reads and identification of single nucleotide polymorphisms

The sequencing run yielded approximately 39 million single-ended reads of 38 bases each. The raw reads were mapped to the reference M. tuberculosis H37Rv genome sequence using Bowtie (Langmead et al, 2009) allowing up to three mismatches and a maximum of 10 different alignments per read. Using these parameters, 98% of the reads could be successfully mapped to the H37Rv genome. Sequence variants were called using the SAMtools pileup function (Li et al, 2009). A total of 191 SNPs were detected and filtered further on the basis of read coverage at the SNP position, resulting in a total of 53 SNPs with a read depth greater than or equal to 100 reads.

Expression and purification of InhA

For the purification of InhA, InhA(S94A) and InhA(D148G), their respective genes were amplified from the pMV261 based vectors described above, and cloned into pDONR by BP recombination cloning followed by LR recombination cloning into pHis9GW vector as has been described previously 35,36 (primer sequences are provided in Supplementary Table 1). The resulting plasmids: pHis9-InhA, pHis9-InhA(S94A) and pHis9-InhA(D148G) were transformed into One Shot® BL21(DE3) Chemically Competent E. coli (Invitrogen). Clones were grown in LB broth to an OD600=0.8, and protein production induced with 500 µM IPTG (isopropyl-β-d-thiogalactopyranoside) overnight (18°C, 200 rpm). Bacteria were pelleted and resuspended in Lysis buffer (50 mM Tris pH7.5, 500 mM NaCl, 1 mM Imidazole, 10% Glycerol, 1% Tween 20) containing DNase, mini-protease inhibitor cocktail tablet (Roche) and lysozyme. Following incubation (1 hr, 4°C), bacteria were disrupted by sonication and cell debris pelleted (30,000 × g, 30 min, 4°C). InhA was then bound to PrepEase resin (His-Tagged Protein Purification Resin-High Specificity supplied by usb corporation) and washed with 50 column volumes of wash buffer (50 mM Tris pH 7.5; 500 mM NaCl; 10 mM Imidazole). Proteins were eluted with 10 column volumes of elution buffer (50 mM Tris pH7, 5; 500 mM NaCl; 250 mM Imidazole), and the purity checked by SDS-PAGE. The pure InhA protein was then dialyzed against 50 mM Tris pH7.5; 300 mM NaCl, concentrated using Amicon Ultra 10kDa concentration columns and used for both in vitro enzyme activity assays (stored with 50% glycerol) or crystal trials.

Synthesis of 2-trans-octenoyl CoA

2-Trans-octenoyl CoA was synthesized using the method described by Kopp et al. (Kopp et al, 2008). The product was purified by preparative HLPC-MS using Method 2 (RT=11.4 min).

Preparative HPLC:

Preparative HPLC was performed on a Waters 2525 HLPC system coupled to a Waters Micromass ZQ detector in ESI+ mode, using a Waters XTerra prep C18 column with particle size 10 µm (18x250 mm), with a flow rate of 20 ml/min, with a gradient using mobile phase A (MPA: 0.1% formic acid, 0.01% TFA in water;) and mobile phase B (MPB: 90% acetonitrile, 0.1% formic acid, 0.01% TFA in water.) For purification of pyridomycin (method 1) a gradient was run from 22% to 35% MPB over 20 min with pyridomycin eluting at 6.4 min. For the purification of 2-trans-octenoyl CoA (method 2) an initial gradient of 0% to 10% MPB was run over 5 min, followed by a gradient from 10% to 100% over 20 min (RT=11.4 min).

Supplementary Figures

Supplementary Figure 1: (a) Superposition of wild-type InhA:NADH (green), InhA(S94A):NADH (green) and InhA(D148G):NADH (blue) structures, with NADH in red. (b) Overlay of the active site catalytic residues of InhA:NADH (green), InhA(S94A):NADH (green) and InhA(D148G):NADH (blue).

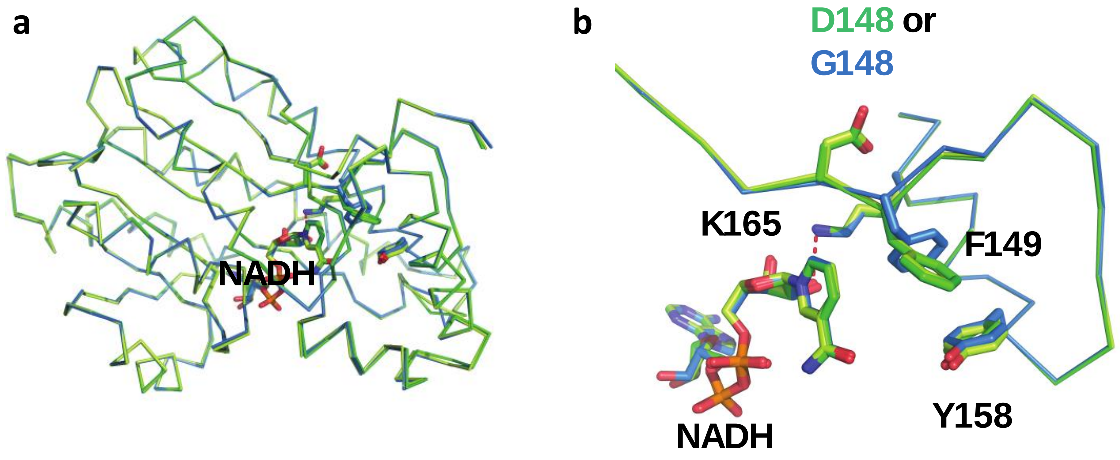

Supplementary Figure 2: Alignment of the InhA protein sequences of various Mycobacteria.

```
               1       10        20        30        40        50        60
               |--------+---------+---------+---------+---------+---------|
TUBERCULOSIS   MTGLLDGKRILVSGIITDSSIAFHIARVAQEQGAQLVLTGFDRLRLIQRITDRLPAKAPL
       BOVIS   MTGLLDGKRILVSGIITDSSIAFHIARVAQEQGAQLVLTGFDRLRLIQRITDRLPAKAPL
     MARINUM   MAGLLEGKRILVSGIITDSSIAFHIARVAQEQGAQLVLTGFDRMRLIQRIVDRLPQKAPL
   ABSCESSUS   MAGLLEGKRILVTGIITDSSIAFHIAKVAQEQGAELVLTGFDRLRLIERITQRLPKPAPL
    BOLLETTI   MAGLLEGKRILVTGIITDSSIAFHIAKVAQEQGAELVLTGFDRLRLIERITQRLPKPAPL
 MASSILIENSE   MAGLLEGKRILVTGIITDSSIAFHIAKVAQEQGAELVLTGFDRLRLIERITQRLPKPAPL
      LEPRAE   MAGLLEGKRILVSGIITDSSIAFHIAKVAQEAGAQLVLTGFDRLRLIQRIADRLPDKAPL
   SMEGMATIS   MTGLLEGKRILVTGIITDSSIAFHIAKVAQEAGAELVLTGFDRLKLVKRIADRLPKPAPL
       AVIUM   MAGLLDGKRILVTGIITDSSIAFHIAKVAQEAGAQLVLTGFDRLRLIQRIVDRLPEKAPL
   Consensus   MaGLL#GKRILVtGIITDSSIAFHIAkVAQEaGA#LVLTGFDR$rL!qRI.#RLP.kAPL

               61      70        80        90        100       110       120
               |--------+---------+---------+---------+---------+---------|
TUBERCULOSIS   LELDVQNEEHLASLAGRVTEAIGAGNKLDGVVHSIGFMPQTGMGINPFFDAPYADVSKGI
       BOVIS   LELDVQNEEHLASLAGRVTEAIGAGNKLDGVVHSIGFMPQTGMGINPFFDAPYADVSKGI
     MARINUM   IELDVQNEEHLASLAGRVTEVIGEGNNLDGVVHSIGFMPQSGMGINPFFDAPYEDVSKGI
   ABSCESSUS   LELDVQNEEHLGSLAGRISEVIGEGNKLDGVVHSIGFMPQSGMGVNPFFDAPFADVSKGF
    BOLLETTI   LELDVQNEEHLDSLAGRVSEAIGEGNKLDGVVHSIGFMPQSGMGVNPFFDAPFADVSKGF
 MASSILIENSE   LELDVQNEEHLDSLAGRVSEAIGEGNKLDGVVHSIGFMPQSGMGVNPFFDAPFADVSKGF
      LEPRAE   IELDVQNEEHLATLAERVTAEIGEGNKLDGVVHSIGFMPQTGMGTNQFFDAPYEDVSKGI
   SMEGMATIS   LELDVQNEEHLSTLADRITAEIGEGNKIDGVVHSIGFMPQSGMGINPFFDAPYEDVSKGI
       AVIUM   IELDVQNEEHLNTLAQRVTAEIGEGNKLDGVVHSIGFMPQTGMGINPFFDAPYEDVSKGI
   Consensus   lELDVQNEEHL.tLA.R!taeIGeGNklDGVVHSIGFMPQsGMGiNpFFDAP%eDVSKGi

               121     130       140       150       160       170       180
               |--------+---------+---------+---------+---------+---------|
TUBERCULOSIS   HISAYSYASMAKALLPIMNPGGSIVGMDFDPSRAMPAYNWMTVAKSALESVNRFVAREAG
       BOVIS   HISAYSYASMAKALLPIMNPGGSIVGMDFDPSRAMPAYNWMTVAKSALESVNRFVAREAG
     MARINUM   HISAYSYASLAKALLPIMNPGGSIVGMDFDPTRAMPAYNWMTVAKSALESVNRFVAREAG
   ABSCESSUS   HISAFSYSSLAKAVLPVMNRGGSIVGMDFDPTRAMPAYNWMTVAKSALESVNRFVAREAG
    BOLLETTI   HISAFSYSSLAKAVLPVMNRGGSIVGMDFDPTRAMPAYNWMTVAKSALESVNRFVAREAG
 MASSILIENSE   HISAFSYSSLAKAVLPVMNRGGSIVGMDFDPTRAMPAYNWMTVAKSALESVNRFVAREAG
      LEPRAE   HISTYSYASLAKALLLIMNSGGSIVGMDFDPTRAMPAYNWMTVAKSALESVNRFVAREAG
   SMEGMATIS   HISAYSYASLAKAVLPIMNPGGGIVGMDFDPTRAMPAYNWMTVAKSALESVNRFVAREAG
       AVIUM   HISAYSYASLAKALLPIMNPGGSIVGMDFDPSRAMPAYNWMTVAKSALESVNRFVAREAG
   Consensus   HISa%SYaS$AKAlLp!MNpGGsIVGMDFDPtRAMPAYNWMTVAKSALESVNRFVAREAG

               181     190       200       210       220       230       240
               |--------+---------+---------+---------+---------+---------|
TUBERCULOSIS   KYGVRSNLVAAGPIRTLAMSAIVGGALGEEAGAQIQLLEEGWDQRAPIGWNMKDATPVAK
       BOVIS   KYGVRSNLVAAGPIRTLAMSAIVGGALGEEAGAQIQLLEEGWDQRAPIGWNMKDATPVAK
     MARINUM   KYGVRSNLVAAGPIRTLAMSAIVGGALGEEAGAQIQLLEDGWDQRAPVGWNMKDPTPVAK
   ABSCESSUS   KVGVRSNLVAAGPIRTLAMSAIVGGALGDEAGQQMQLLEEGWDQRAPIGWDMKDPTPVAK
    BOLLETTI   KVGVRSNLVAAGPIRTLAMSAIVGGALGDEAGQQMQLLEEGWDQRAPIGWDMKDPTPVAK
 MASSILIENSE   KVGVRSNLVAAGPIRTLAMSAIVGGALGDEAGQQMQLLEEGWDQRAPIGWDMKDPTPVAK
      LEPRAE   KYGVRSNLVAAGPIRTLAMSAIVGGAFGEEAGAQMQLLEEGWDQRAPIGWNMKDPTPVAK
   SMEGMATIS   KVGVRSNLVAAGPIRTLAMSAIVGGALGDEAGQQMQLLEEGWDQRAPLGWNMKDPTPVAK
       AVIUM   PHGVRSNLVAAGPIRTLAMAGIVGGVLGDQAAEQIRLLEEGWDQRAPIGWNMKDPTPVAK
   Consensus   k.GVRSNLVAAGPIRTLAMsaIVGGalG##Ag.QmqLLE#GWDQRAPiGW#MKDpTPVAK

               241     250       260     269
               |--------+---------+--------|
TUBERCULOSIS   TVCALLSDWLPATTGDIIYADGGAHTQLL
       BOVIS   TVCALLSDWLPATTGDIIYADGGAHTQLL
     MARINUM   TVCAVLSEWLPATTGDIIFADGGAHTQLL
   ABSCESSUS   TVCALLSDWLPATTGDIIFADGGAHTQLL
    BOLLETTI   TVCALLSDWLPATTGDIIFADGGAHTQLL
 MASSILIENSE   TVCALLSDWLPATTGDIIFADGGAHTQLL
      LEPRAE   TVCALLSEWLPATTGSIIYADGGASTQLL
   SMEGMATIS   TVCALLSDWLPATTGTVIYADGGASTQLL
       AVIUM   TVCALLSDWLPATTGTIIYADGGASTQLL
   Consensus   TVCAlLS#WLPATTG.!I%ADGGAsTQLL
```

Supplementary tables

Supplementary table 1: Primers used in manuscript.

Primers for InhA amplification and Gateway cloning

PCR1	InhA_Nthr:	CTGGTTCCGCGTGGATCCACAGGACTGCTGGACGGCAAACGG
	InhA_Cstop:	CAAGAAAGCTGGGTCTCAGAGCAATTGGGTGTGCGCGC
PCR2	attB1-thrombin	GGGGACAAGTTTGTACAAAAAAGCAGGCTTCCTGGTTCCGCGTGGATC
	attB2	GGGGACCACTTTGTACAAGAAAGCTGGGTC

Primers for site directed mutagenesis

t280g_sense_(S94A)	CGA CGG GGT GGT GCA TGC GAT TGG GTT CA
t280g_antisense_(S94A)	TGA ACC CAA TCG CAT GCA CCA CCC CGT CG
a443g_sense_(D148G)	ATC GTC GGC ATG GGC TTC GAC CCG AGC
a443g_antisense_(D148G)	GCT CGG GTC GAA GCC CAT GCC GAC GAT

Supplementary table 2: Data collection and refinement statistics (Molecular Replacement)

	**InhA**	**InhA(S94A)**	**InhA(D148G)**
**Data collection**			
Space group	P6222	P6222	P6222
Cell dimensions			
a, *b*, *c* (Å)	97.65, 97.65, 139.56	97.99, 97.99, 139.60	98.45, 98.45, 139.21
α, β, γ (°)	90, 90, 120	90, 90, 120	90, 90, 120
Resolution (Å)	50-2.39(2.54-2.39)	50-1.90 (2.02-1.90)	50.0-2.45 (2.6-2.45)
R_{meas}	11.8 (67.4)	7.0 (69.0)	7.0 (47.4)
$I / \sigma I$	13.29 (2.60)	19.60 (2.73)	35.8 (7.5)
Completeness (%)	99.1 (95.7)	90.8 (92.3)	99.8 (99.4)
Redundancy	20.47 (5.78)	6.80 (5.99)	20.5 (20.47)
**Refinement**			
Resolution (Å)	2.40	1.90	2.45
No. reflections	15218	27184	14464
R_{work} / R_{free}	0.19/0.25	0.19/0.21	0.21/0.25
No. atoms			
Protein	1985	1993	1985
Ligand	44	44	44
Water	38	146	47
B-factor	32.6	24.9	36.8
R.m.s deviations			
Bond lengths (Å)	0.018	0.010	0.018
Bond angles (°)	2.207	1.585	2.20
PDB code	4DRE	4DTI	4DQU

One crystal for each structure. Highest resolution shell is shown in parenthesis.

References

Kinoshita M, Nakata M, Takarada K, Tatsuta K (1989) Synthetic Studies of Pyridomycin .5. Total Synthesis of Pyridomycin. *Tetrahedron Lett* **30:** 7419-7422

Kopp F, Linne U, Oberthur M, Marahiel MA (2008) Harnessing the chemical activation inherent to carrier protein-bound thioesters for the characterization of lipopeptide fatty acid tailoring enzymes. *J Am Chem Soc* **130:** 2656-2666

Langmead B, Trapnell C, Pop M, Salzberg SL (2009) Ultrafast and memory-efficient alignment of short DNA sequences to the human genome. *Genome Biol* **10:** R25

Li H, Handsaker B, Wysoker A, Fennell T, Ruan J, Homer N, Marth G, Abecasis G, Durbin R (2009) The Sequence Alignment/Map format and SAMtools. *Bioinformatics* **25:** 2078-2079
